# 2-(2-Meth­oxy­phen­yl)-1*H*-isoindole-1,3(2*H*)-dione

**DOI:** 10.1107/S1600536812027262

**Published:** 2012-07-28

**Authors:** M. Nawaz Tahir, Muhammad Sirajuddin, Saqib Ali, Khurram Shahzad Munawar

**Affiliations:** a Department of Physics, University of Sargodha, Sargodha, Pakistan; b Department of Chemistry, Quaid-i-Azam University, Islamabad, Pakistan

## Abstract

In the title compound, C_15_H_11_NO_3_, the dihedral angle between the meth­oxy­benzene and isoindole ring systems is 70.21 (3)°. The meth­oxy C atom is close to being coplanar with its attached ring [deviation = 0.133 (2) Å] and is oriented away from the isoindole moiety. In the crystal, inversion dimers linked by pairs of C—H⋯O hydrogen bonds generate *R*_2_^2^(10) loops. Further C—H⋯O inter­actions lead to (010) infinite sheets and weak aromatic π–π stacking [centroid–centroid separations = 3.6990 (10) and 3.7217 (10) Å] is also observed.

## Related literature

For related structures, see: Sim *et al.* (2009 ▶); Sirajuddin *et al.* (2012 ▶). For hydrogen-bond motifs, see: Bernstein *et al.*, 1995) ▶.
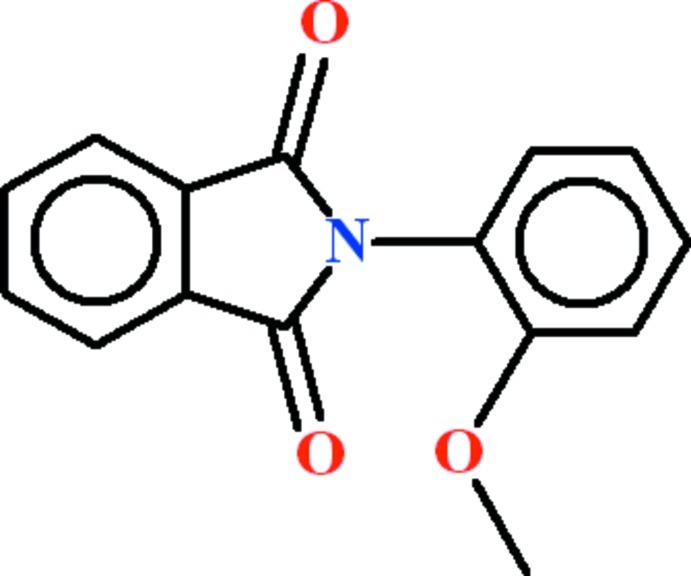


## Experimental

### 

#### Crystal data

C_15_H_11_NO_3_
*M* *_r_* = 253.25
Orthorhombic, 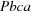

*a* = 11.5768 (6) Å
*b* = 7.3222 (5) Å
*c* = 29.2849 (15) Å
*V* = 2482.4 (2) Å^3^
*Z* = 8
Mo *K*α radiation
μ = 0.10 mm^−1^
*T* = 296 K
0.32 × 0.26 × 0.24 mm

#### Data collection

Bruker Kappa APEXII CCD diffractometer
Absorption correction: multi-scan (*SADABS*; Bruker, 2005 ▶) *T*_min_ = 0.969, *T*_max_ = 0.977
10815 measured reflections
2428 independent reflections
1816 reflections with *I* > 2σ(*I*)
*R*_int_ = 0.024

#### Refinement

*R*[*F* ^2^ > 2σ(*F* ^2^)] = 0.041
*wR*(*F* ^2^) = 0.112
*S* = 1.03
2428 reflections
173 parameters
H-atom parameters constrained
Δρ_max_ = 0.12 e Å^−3^
Δρ_min_ = −0.17 e Å^−3^

### 

Data collection: *APEX2* (Bruker, 2007 ▶); cell refinement: *SAINT* (Bruker, 2007 ▶); data reduction: *SAINT*; program(s) used to solve structure: *SHELXS97* (Sheldrick, 2008 ▶); program(s) used to refine structure: *SHELXL97* (Sheldrick, 2008 ▶); molecular graphics: *ORTEP-3 for Windows* (Farrugia, 1997 ▶) and *PLATON* (Spek, 2009 ▶); software used to prepare material for publication: *WinGX* (Farrugia, 1999 ▶) and *PLATON*.

## Supplementary Material

Crystal structure: contains datablock(s) global, I. DOI: 10.1107/S1600536812027262/hb6857sup1.cif

Structure factors: contains datablock(s) I. DOI: 10.1107/S1600536812027262/hb6857Isup2.hkl

Supplementary material file. DOI: 10.1107/S1600536812027262/hb6857Isup3.cml

Additional supplementary materials:  crystallographic information; 3D view; checkCIF report

## Figures and Tables

**Table 1:** Hydrogen-bond geometry (Å, °)

*D*—H⋯*A*	*D*—H	H⋯*A*	*D*⋯*A*	*D*—H⋯*A*
C3—H3⋯O1^i^	0.93	2.57	3.428 (2)	153
C12—H12⋯O2^ii^	0.93	2.46	3.313 (2)	152

Symmetry codes: (i) 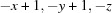; (ii) 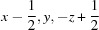.

**Fig. 1.**
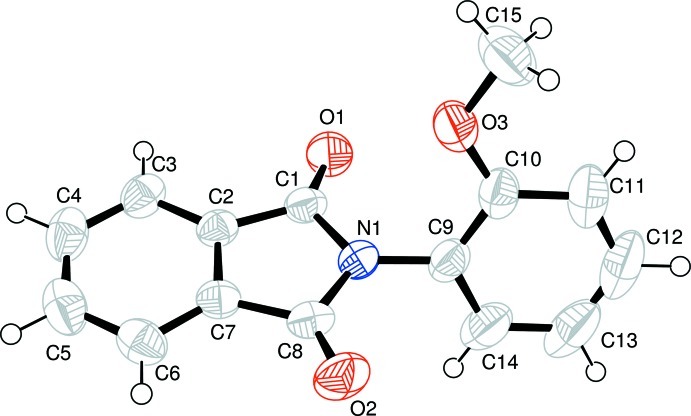
View of the title compound with displacement ellipsoids drawn at the 50% probability level.

**Fig. 2.**
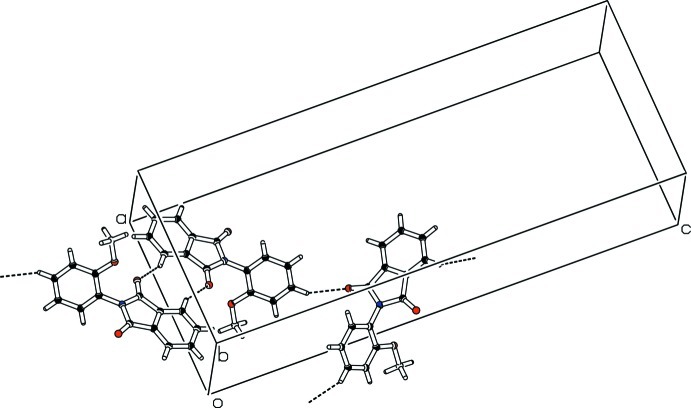
The partial packing, which shows that molecules form dimers with R22(10) ring mtifs and C(18) chains are formed due to C—H···O bonds.

## supplementary crystallographic information

### Comment

The title compound (I), (Fig. 1) has been synthesized in an attempt to form the carboxylic acid containing methoxybenzene. We have reported the crystal structure of 1-(2-methoxyphenyl)-1*H*-pyrrole-2,5-dione (Sirajuddin *et al.*, 2012) which is related to (I). The polymorph of (I) has also been published by (Sim *et al.*, 2009).

In (I), 1*H*-isoindole-1,3(2*H*)-dione A (C1—C8/N1/O1/O2) and the methoxybenzene B (C9—C15/O3) are almost planar with r.m.s. deviation of 0.0458 and 0.0320 Å, respectively. The dihedral angle between A/B is 70.21 (3)°. The molecules are dimerized due to C—H···O type of H-bonding with *R*_2_^2^(10) ring motifs (Bernstein *et al.*, 1995). The dimers are interlinked due to further C—H···O bonds to form infinite sheets. There exist π···π interaction between *Cg*1···*Cg*2^i^ [i = 3/2 - *x*, -1/2 + *y*, *z*] and *Cg*2···*Cg*1^ii^ [ii = 3/2 - *x*, 1/2 + *y*, *z*] at a distance of 3.7217 (10) Å. Similarly, there exist π···π interaction between *Cg*2··· *Cg*2^i^ [i = 3/2 - *x*, -1/2 + *y*, *z*] and *Cg*2··· *Cg*2^ii^ [ii = 3/2 - *x*, 1/2 + *y*, *z*] at a distance of 3.6990 (10) Å. *Cg*1 and *Cg*2 are the centroids of (C1/C2/C7/C8/N1) and (C2—C7) rings, respectively.

### Experimental

Equimolar quantities of 2-methoxyaniline and phthalic anhydride were stirred and refluxed in acetic acid for 4 h. The solution was kept at room temperature which afforded dark yellow prisms after 12 h.

### Refinement

The H-atoms were positioned geometrically (C–H = 0.93–0.96 Å) and refined as riding with *U*_iso_(H) = *xU*_eq_(C), where *x* = 1.5 for methyl and *x* = 1.2 for other H-atoms.

### Crystal data

**Table:**

C_15_H_11_NO_3_	*F*(000) = 1056
*M**_r_* = 253.25	*D*_x_ = 1.355 Mg m^−^^3^
Orthorhombic, *P**b**c**a*	Mo *K*α radiation, λ = 0.71073 Å
Hall symbol: -P 2ac 2ab	Cell parameters from 1816 reflections
*a* = 11.5768 (6) Å	θ = 2.2–26.0°
*b* = 7.3222 (5) Å	µ = 0.10 mm^−^^1^
*c* = 29.2849 (15) Å	*T* = 296 K
*V* = 2482.4 (2) Å^3^	Prism, dark yellow
*Z* = 8	0.32 × 0.26 × 0.24 mm

### Data collection

**Table:**

Bruker Kappa APEXII CCD diffractometer	2428 independent reflections
Radiation source: fine-focus sealed tube	1816 reflections with *I* > 2σ(*I*)
Graphite monochromator	*R*_int_ = 0.024
Detector resolution: 8.00 pixels mm^-1^	θ_max_ = 26.0°, θ_min_ = 2.2°
ω scans	*h* = −14→14
Absorption correction: multi-scan (*SADABS*; Bruker, 2005)	*k* = −9→5
*T*_min_ = 0.969, *T*_max_ = 0.977	*l* = −35→36
10815 measured reflections	

### Refinement

**Table:**

Refinement on *F*^2^	Primary atom site location: structure-invariant direct methods
Least-squares matrix: full	Secondary atom site location: difference Fourier map
*R*[*F*^2^ > 2σ(*F*^2^)] = 0.041	Hydrogen site location: inferred from neighbouring sites
*wR*(*F*^2^) = 0.112	H-atom parameters constrained
*S* = 1.03	*w* = 1/[σ^2^(*F*_o_^2^) + (0.0502*P*)^2^ + 0.6208*P*] where *P* = (*F*_o_^2^ + 2*F*_c_^2^)/3
2428 reflections	(Δ/σ)_max_ < 0.001
173 parameters	Δρ_max_ = 0.12 e Å^−^^3^
0 restraints	Δρ_min_ = −0.17 e Å^−^^3^

### Special details

**Table:**

Geometry. Bond distances, angles *etc*. have been calculated using the rounded fractional coordinates. All su's are estimated from the variances of the (full) variance-covariance matrix. The cell e.s.d.'s are taken into account in the estimation of distances, angles and torsion angles
Refinement. Refinement of *F*^2^ against ALL reflections. The weighted *R*-factor *wR* and goodness of fit *S* are based on *F*^2^, conventional *R*-factors *R* are based on *F*, with *F* set to zero for negative *F*^2^. The threshold expression of *F*^2^ > σ(*F*^2^) is used only for calculating *R*-factors(gt) *etc*. and is not relevant to the choice of reflections for refinement. *R*-factors based on *F*^2^ are statistically about twice as large as those based on *F*, and *R*- factors based on ALL data will be even larger.

### Fractional atomic coordinates and isotropic or equivalent isotropic displacement parameters (Å^2^)

**Table:**

	*x*	*y*	*z*	*U*_iso_*/*U*_eq_	
O1	0.45483 (10)	0.37357 (19)	0.07121 (4)	0.0609 (4)	
O2	0.74031 (12)	0.1478 (2)	0.16275 (4)	0.0672 (5)	
O3	0.41409 (11)	−0.01341 (19)	0.11923 (4)	0.0632 (5)	
N1	0.57642 (11)	0.2519 (2)	0.12589 (4)	0.0451 (4)	
C1	0.54983 (14)	0.3245 (2)	0.08300 (5)	0.0432 (5)	
C2	0.66018 (14)	0.3329 (2)	0.05763 (5)	0.0409 (5)	
C3	0.68405 (17)	0.3932 (2)	0.01415 (6)	0.0512 (6)	
C4	0.79860 (18)	0.3945 (3)	0.00060 (6)	0.0607 (6)	
C5	0.88538 (17)	0.3416 (3)	0.02983 (7)	0.0635 (7)	
C6	0.86140 (15)	0.2806 (2)	0.07348 (7)	0.0551 (6)	
C7	0.74722 (14)	0.2752 (2)	0.08649 (5)	0.0422 (5)	
C8	0.69509 (14)	0.2162 (2)	0.13013 (5)	0.0454 (5)	
C9	0.49518 (15)	0.2254 (3)	0.16170 (5)	0.0501 (5)	
C10	0.41319 (15)	0.0874 (3)	0.15822 (6)	0.0530 (6)	
C11	0.33615 (18)	0.0624 (3)	0.19381 (7)	0.0716 (8)	
C12	0.3427 (2)	0.1736 (4)	0.23180 (7)	0.0881 (9)	
C13	0.4240 (2)	0.3075 (4)	0.23528 (7)	0.0922 (10)	
C14	0.5005 (2)	0.3346 (3)	0.19983 (6)	0.0724 (8)	
C15	0.3388 (2)	−0.1661 (3)	0.11653 (9)	0.0873 (10)	
H3	0.62552	0.43151	−0.00537	0.0615*	
H4	0.81725	0.43190	−0.02884	0.0728*	
H5	0.96171	0.34706	0.02003	0.0762*	
H6	0.92002	0.24465	0.09325	0.0661*	
H11	0.28034	−0.02875	0.19216	0.0860*	
H12	0.29047	0.15681	0.25555	0.1058*	
H13	0.42784	0.37989	0.26133	0.1107*	
H14	0.55562	0.42669	0.20171	0.0869*	
H15A	0.35417	−0.24768	0.14146	0.1309*	
H15B	0.35107	−0.22876	0.08815	0.1309*	
H15C	0.26011	−0.12507	0.11816	0.1309*	

### Atomic displacement parameters (Å^2^)

**Table:**

	*U*^11^	*U*^22^	*U*^33^	*U*^12^	*U*^13^	*U*^23^
O1	0.0466 (7)	0.0825 (9)	0.0537 (7)	0.0051 (6)	−0.0098 (6)	0.0129 (6)
O2	0.0672 (8)	0.0852 (10)	0.0493 (7)	0.0108 (7)	−0.0175 (6)	0.0115 (7)
O3	0.0594 (8)	0.0663 (8)	0.0640 (8)	−0.0159 (7)	0.0127 (6)	−0.0053 (7)
N1	0.0446 (7)	0.0554 (8)	0.0354 (7)	−0.0026 (6)	−0.0048 (6)	0.0049 (6)
C1	0.0469 (9)	0.0450 (9)	0.0377 (8)	−0.0029 (7)	−0.0084 (7)	0.0011 (7)
C2	0.0486 (9)	0.0354 (8)	0.0386 (8)	−0.0039 (7)	−0.0041 (7)	−0.0020 (7)
C3	0.0687 (11)	0.0425 (9)	0.0424 (9)	−0.0034 (8)	−0.0003 (8)	0.0020 (7)
C4	0.0791 (13)	0.0484 (10)	0.0546 (10)	−0.0081 (10)	0.0193 (10)	0.0008 (9)
C5	0.0580 (11)	0.0530 (11)	0.0794 (14)	−0.0071 (9)	0.0204 (10)	−0.0039 (10)
C6	0.0472 (10)	0.0479 (10)	0.0701 (12)	−0.0016 (8)	−0.0024 (9)	−0.0064 (9)
C7	0.0466 (9)	0.0350 (8)	0.0451 (9)	−0.0031 (7)	−0.0037 (7)	−0.0049 (7)
C8	0.0497 (9)	0.0450 (9)	0.0414 (9)	−0.0003 (7)	−0.0116 (7)	−0.0016 (7)
C9	0.0507 (9)	0.0640 (11)	0.0356 (8)	0.0064 (9)	−0.0014 (7)	0.0044 (8)
C10	0.0499 (10)	0.0628 (11)	0.0464 (9)	0.0077 (9)	0.0064 (8)	0.0125 (9)
C11	0.0632 (12)	0.0882 (16)	0.0635 (12)	0.0137 (11)	0.0195 (10)	0.0266 (12)
C12	0.0832 (16)	0.131 (2)	0.0502 (12)	0.0392 (16)	0.0234 (12)	0.0282 (14)
C13	0.1046 (19)	0.131 (2)	0.0410 (11)	0.0330 (18)	0.0029 (12)	−0.0085 (13)
C14	0.0815 (14)	0.0915 (16)	0.0442 (10)	0.0069 (12)	−0.0051 (10)	−0.0098 (10)
C15	0.0696 (14)	0.0819 (16)	0.1103 (19)	−0.0268 (12)	0.0106 (13)	−0.0047 (14)

### Geometric parameters (Å, º)

**Table:**

O1—C1	1.207 (2)	C9—C14	1.375 (3)
O2—C8	1.199 (2)	C10—C11	1.384 (3)
O3—C10	1.360 (2)	C11—C12	1.381 (3)
O3—C15	1.420 (3)	C12—C13	1.363 (4)
N1—C1	1.3982 (19)	C13—C14	1.379 (3)
N1—C8	1.404 (2)	C3—H3	0.9300
N1—C9	1.422 (2)	C4—H4	0.9300
C1—C2	1.479 (2)	C5—H5	0.9300
C2—C3	1.376 (2)	C6—H6	0.9300
C2—C7	1.381 (2)	C11—H11	0.9300
C3—C4	1.384 (3)	C12—H12	0.9300
C4—C5	1.376 (3)	C13—H13	0.9300
C5—C6	1.382 (3)	C14—H14	0.9300
C6—C7	1.376 (2)	C15—H15A	0.9600
C7—C8	1.478 (2)	C15—H15B	0.9600
C9—C10	1.390 (3)	C15—H15C	0.9600
			
C10—O3—C15	118.01 (16)	C10—C11—C12	119.6 (2)
C1—N1—C8	111.45 (12)	C11—C12—C13	121.5 (2)
C1—N1—C9	124.67 (13)	C12—C13—C14	119.4 (2)
C8—N1—C9	123.82 (13)	C9—C14—C13	119.9 (2)
O1—C1—N1	124.79 (14)	C2—C3—H3	121.00
O1—C1—C2	129.10 (14)	C4—C3—H3	121.00
N1—C1—C2	106.07 (13)	C3—C4—H4	119.00
C1—C2—C3	130.67 (15)	C5—C4—H4	119.00
C1—C2—C7	108.06 (13)	C4—C5—H5	119.00
C3—C2—C7	121.19 (16)	C6—C5—H5	119.00
C2—C3—C4	117.39 (17)	C5—C6—H6	121.00
C3—C4—C5	121.29 (17)	C7—C6—H6	121.00
C4—C5—C6	121.32 (18)	C10—C11—H11	120.00
C5—C6—C7	117.27 (17)	C12—C11—H11	120.00
C2—C7—C6	121.50 (15)	C11—C12—H12	119.00
C2—C7—C8	108.70 (14)	C13—C12—H12	119.00
C6—C7—C8	129.79 (15)	C12—C13—H13	120.00
O2—C8—N1	125.14 (15)	C14—C13—H13	120.00
O2—C8—C7	129.26 (15)	C9—C14—H14	120.00
N1—C8—C7	105.59 (12)	C13—C14—H14	120.00
N1—C9—C10	119.79 (15)	O3—C15—H15A	110.00
N1—C9—C14	119.34 (18)	O3—C15—H15B	109.00
C10—C9—C14	120.86 (16)	O3—C15—H15C	109.00
O3—C10—C9	116.81 (15)	H15A—C15—H15B	109.00
O3—C10—C11	124.44 (18)	H15A—C15—H15C	109.00
C9—C10—C11	118.75 (17)	H15B—C15—H15C	109.00
			
C15—O3—C10—C9	174.12 (17)	C3—C2—C7—C6	1.9 (2)
C15—O3—C10—C11	−6.5 (3)	C3—C2—C7—C8	−179.07 (14)
C8—N1—C1—O1	−177.05 (15)	C2—C3—C4—C5	−1.5 (3)
C8—N1—C1—C2	0.97 (17)	C3—C4—C5—C6	1.7 (3)
C9—N1—C1—O1	0.1 (3)	C4—C5—C6—C7	−0.1 (3)
C9—N1—C1—C2	178.09 (16)	C5—C6—C7—C2	−1.7 (2)
C1—N1—C8—O2	−177.22 (15)	C5—C6—C7—C8	179.52 (17)
C1—N1—C8—C7	1.25 (17)	C2—C7—C8—O2	175.22 (16)
C9—N1—C8—O2	5.6 (3)	C2—C7—C8—N1	−3.16 (16)
C9—N1—C8—C7	−175.90 (15)	C6—C7—C8—O2	−5.9 (3)
C1—N1—C9—C10	71.9 (2)	C6—C7—C8—N1	175.74 (15)
C1—N1—C9—C14	−109.5 (2)	N1—C9—C10—O3	−1.6 (3)
C8—N1—C9—C10	−111.38 (19)	N1—C9—C10—C11	179.02 (17)
C8—N1—C9—C14	67.2 (3)	C14—C9—C10—O3	179.80 (18)
O1—C1—C2—C3	−1.9 (3)	C14—C9—C10—C11	0.4 (3)
O1—C1—C2—C7	174.92 (16)	N1—C9—C14—C13	−178.45 (19)
N1—C1—C2—C3	−179.77 (16)	C10—C9—C14—C13	0.2 (3)
N1—C1—C2—C7	−2.98 (16)	O3—C10—C11—C12	−179.6 (2)
C1—C2—C3—C4	176.14 (17)	C9—C10—C11—C12	−0.3 (3)
C7—C2—C3—C4	−0.3 (2)	C10—C11—C12—C13	−0.4 (4)
C1—C2—C7—C6	−175.24 (14)	C11—C12—C13—C14	1.0 (4)
C1—C2—C7—C8	3.77 (17)	C12—C13—C14—C9	−0.8 (4)

### Hydrogen-bond geometry (Å, º)

**Table:**

*D*—H···*A*	*D*—H	H···*A*	*D*···*A*	*D*—H···*A*
C3—H3···O1^i^	0.93	2.57	3.428 (2)	153
C12—H12···O2^ii^	0.93	2.46	3.313 (2)	152

Symmetry codes: (i) −*x*+1, −*y*+1, −*z*; (ii) *x*−1/2, *y*, −*z*+1/2.
